# Hypoparathyroidism: clinical profiles, healthcare use, and costs from real-world data from Italy

**DOI:** 10.1530/EC-25-0844

**Published:** 2026-03-26

**Authors:** Andrea Palermo, Guido Zavatta, Erica Solaroli, Letizia Dondi, Nicola Ambrosio, Leonardo Dondi, Giulia Ronconi, Irene Dell’Anno, Alice Addesi, Immacolata Esposito, Anna Piazza, Nello Martini, Carlo Piccinni

**Affiliations:** ^1^ Unit of Metabolic Bone and Thyroid Disorders, Fondazione Policlinico Universitario Campus Bio-Medico, Rome, Italy; ^2^ Division of Endocrinology and Diabetes Prevention and Care, IRCCS Azienda Ospedaliero-Universitaria di Bologna, Bologna, Italy; ^3^ Department of Medical and Surgical Sciences (DIMEC), Alma Mater Studiorum University of Bologna, Bologna, Italy; ^4^ Unit of Endocrinology, Department of Medicine, AUSL Bologna, Bologna, Italy; ^5^ Fondazione Ricerca e Salute (ReS) – Research and Health Foundation, Rome, Italy; ^6^ Drugs and Health Srl, Rome, Italy

**Keywords:** administrative database, hypoparathyroidism, epidemiology, healthcare resources, hypocalcemia therapy

## Abstract

**Aim:**

To describe the epidemiological, clinical, and healthcare profiles of treated hypoparathyroidism in Italy and to evaluate healthcare resource utilization and direct costs.

**Methods:**

Patients with hypoparathyroidism between 2016 and 2022 were identified from the ReS administrative database (∼5.5 million Italian citizens of various regions) through an algorithm based on exemption codes, pharmaceutical consumption, hospital diagnosis/procedures, and emergency department (ED) access. The disease prevalence was determined. Demography, clinical characteristics, 1-year healthcare resource utilization (pharmaceuticals, hospitalizations, ED accesses, and outpatient services), and direct costs were analyzed for the cohort of treated patients and for a sub-cohort with higher treatment intensity (HTI) (i.e. with high-dose of calcium/calcitriol).

**Results:**

Hypoparathyroidism’s annual prevalence ranged from 25.5 to 27.6 per 100,000, with a higher prevalence among middle-aged females. The treated patient cohort (*n* = 2,791) and the sub-cohort with HTI (*n* = 662) were identified. Among treated patients, 75.2% had at least one comorbidity and 22.2% had three or more; these percentages were higher in the sub-cohort. A widespread consumption of calcium and/or vitamin D was observed in both cohorts, whereas other treatments were scarcely used. Hospitalization and ED access rates were 24.2 and 24.7%, respectively, for the main cohort and 37.8 and 32.5%, respectively, for the sub-cohort. Mean annual per-patient costs were €2,885 for the main cohort and €3,791 for the sub-cohort.

**Conclusion:**

Hypoparathyroidism, due to its chronicity and complex comorbidity profile, represents a substantial and long-term source of healthcare expenditure. These findings highlight an unmet need, particularly for HTI patients, requiring more specific therapies to improve outcomes.

## Introduction

Hypoparathyroidism is a rare endocrine disorder characterized by a deficient or absent parathyroid hormone (PTH) production or activity, resulting in various multisystem complications as a consequence of dysregulated calcium and phosphate homeostasis (1, 2). Insufficient plasma PTH levels precipitate in a broad range of acute or chronic systemic complications impacting the renal, cardiovascular, neurological, skeletal, and immune systems (3). Clinically, patients frequently report symptoms such as muscle pain, cramps, paresthesia, anxiety, or gastrointestinal disorders (4). Furthermore, chronic hypocalcemia confers an increased risk of severe long-term morbidities, including cataracts, basal ganglia calcifications, ischemic cardiac diseases, cardiac arrhythmias, renal insufficiency, and depression (3, 5, 6, 7).

Etiologically, cases are broadly categorized as surgical and nonsurgical. Postsurgical hypoparathyroidism is a common complication of thyroid surgery or radical neck dissections, typically arising from the inadvertent removal or devascularization of the parathyroid (3, 8, 9).

Actual conventional therapy is based on calcium supplements and active vitamin D analogs, although required doses vary widely and a substantial proportion of patients do not require high-dose calcium supplementation, reflecting heterogeneity in real-world management, while thiazides diuretics are often used to mitigate hypercalciuria (10, 11). Recent European Society of Endocrinology (ESE) guidelines on chronic hypoparathyroidism (12) emphasize individualized titration of calcium and active vitamin D to maintain biochemical control, careful monitoring of urinary calcium, and avoidance of overtreatment whenever possible. However, conventional regimen merely corrects hypocalcemia without replacing the physiological activity and concentration of the physiological parathyroid hormone (3, 13). Furthermore, a high dose of oral calcium salts and vitamin D may exacerbate hyperphosphatemia and hypercalciuria, thereby increasing the risk of long-term renal complications (14). Consequently, the need for a more effective and safer therapeutic strategy has been well recognized. In response, treatment paradigms are shifting toward PTH analogs, which aim to restore physiological control and improve long-term patient outcomes (15, 16). Recent ESE guidelines also provide guidance on PTH replacement therapy (12).

Although hypoparathyroidism is a rare diagnosis, its chronic nature has been widely documented (17); therefore, a substantial number of individuals are living with its long-term clinical consequences and there is a considerable financial burden on healthcare systems (18).

The findings of epidemiological studies indicate an annual incidence ranging from 0.8 to 2.6 per 100,000 individuals; conversely, the prevalence of the disease is found to be higher, ranging from 22 to 37 per 100,000 individuals, thereby underscoring the chronic nature of the condition (19, 20). This pattern is observed worldwide, with variations by region: an incidence of 0.8 and a prevalence of 24 per 100,000 have been reported in Denmark, while data from India and the United States showed an incidence of 2.6 and a prevalence of 37 per 100,000, respectively (21, 22, 23). In line with those data, a study in Italy further confirmed these figures, reporting a prevalence of 27 per 100,000 (24). To date, there have been few epidemiological studies on this disease and even fewer based on real-world data.

This study aims to characterize the population of patients with treated hypoparathyroidism using real-world data from an Italian administrative healthcare database. Our primary objective is to describe the epidemiological, clinical, and healthcare profiles of treated hypoparathyroidism in Italy and to evaluate healthcare resource utilization and direct costs charged by the Italian National Health Service (Servizio Sanitario Nazionale – SSN). A secondary objective is to characterize a sub-cohort of patients with higher treatment intensity (HTI), to identify potential unmet clinical need and the associated economic burden.

## Methods

### Data sources

This retrospective observational study utilized secondary data from the Fondazione Ricerca e Salute database (ReS DB) (25). This database integrates administrative healthcare data annually reported by local and regional healthcare authorities to the Italian Ministry of Health, upon specific agreements for reimbursement purposes, in line with the universal coverage characteristic of the Italian SSN.

ReS DB collects data on exemptions, hospitalizations (diagnosis and procedures), access to emergency department (ED), and drug dispensations for beneficiaries of the Italian SSN, which is universalistic (i.e., covers all citizens).

The study employed data from 2014 to 2022, covering approximately 5 million inhabitants annually. This cohort represents roughly 9% of the Italian population and is broadly representative of its demographic composition (Fig. S1 in Supplementary material (see section on Supplementary materials given at the end of the article)) (26). Data used were anonymized at the source and analyzed in aggregated form according to European Regulations 2014/536 (27) and 2016/679 (28), to which the regional/local authorities, owners of the data, have agreed. Informed consent was waived according to the specific Italian Privacy Authority’s provision (29). Ethical approval was waived according to the European Regulation 2014/536, and it is not expected by the most recent national legislation on observational studies, which requires the ethics committee’s positive opinion only for prospective observational studies, while it does not refer to retrospective ones.

### Study design and cohort’s selection

The study cohort was identified from the overall ReS population, including children, observed in the database from 2016 to 2022 (the accrual period), requiring at least one year of database history prior to identification. Patients with an administrative diagnosis of chronic hypoparathyroidism were selected, with the index date defined as the first recorded evidence of the condition. Case identification has been made according to at least one of the following four criteria (the full list of codes used for each criterion is reported in Table S1 in supplementary material):

- an active exemption of hypoparathyroidism,
- a hospital admission with diagnosis of hypoparathyroidism (International Classification of Diseases, 9th revision – Clinical Modification – ICD-9 CM code 252.1),
- a hospitalization for anterior neck surgery followed by at least one dispensation of calcium within the following 6 months and refilled between 12 and 24 months after the surgery, or
- the presence of rare syndromes related to hypoparathyroidism associated with four or more dispensations of calcium in the subsequent year.

Following the identification of patients with hypoparathyroidism in accordance with the aforementioned criteria, the cohort of treated patients was selected based on the presence of at least one drug dispensation for hypoparathyroidism treatment (i.e. calcium, teriparatide, and vitamin D and analogs; the full list of Anatomical Therapeutic Chemical (ATC) codes is reported in Table S2). Patients affected by hypoparathyroidism and those in treatment were observed annually to analyze trends in prevalence, expressed per 100,000 inhabitants.

Furthermore, a sub-cohort of patients with HTI has been defined as those who met at least one of the following criteria:

- a daily average consumption of supplementary elemental calcium ≥2,000 mg,
- a daily average consumption of calcitriol ≥1 μg,
- a recorded visit for plasma calcium level testing ≥5 during the 12-month follow-up observation period, or
- a teriparatide dispensation ≥1 during the 12-month follow-up period.

Both ‘cohort of treated hypoparathyroidism’ and HTI were characterized in terms of demographic profile, baseline comorbidities, healthcare resource utilization, and associated costs.

### Baseline characteristics

The selected patients of the cohort (i.e. treated hypoparathyroidism) and the sub-cohort (i.e. HTI hypoparathyroidism) have been described at the index date in terms of sex, age, and comorbidities of interest (arterial hypertension, dyslipidemia, thyroid diseases, diabetes, neoplasia, depression, coronary artery disease, cardiac arrhythmias, chronic lung disease, chronic liver diseases, cerebrovascular diseases, heart failure, and chronic kidney disease – identification criteria in Table S3). Furthermore, patients with active or prior history of neoplasia were further analyzed to provide the absolute number and percentage of subjects with thyroid cancer. These subjects were identified based on at least one of the following criteria: a diagnosis of malignant neoplasm of the thyroid gland (ICD-9-CM 193), a personal history of thyroid carcinoma (ICD-9-CM V10.87), or a dispensation of thyrotropin alfa (ATC V04CJ01).

### Healthcare resource consumption and costs

Healthcare resource utilization and associated costs were evaluated over a 12-month follow-up period. Pharmaceutical dispensations of drugs for hypoparathyroidism were analyzed, in terms of absolute dispensations and the number and percentage of patients treated relative to the total cohort stratified by frequency and therapeutic class, along with the average consumption in defined daily doses. In addition, other drugs, such as drugs for hyperphosphatemia, magnesium, and thiazide diuretics (ATC codes are reported in Table S4), were analyzed to complete the therapeutic framework of hypoparathyroidism. Hospitalizations and ED visits were analyzed by calculating the absolute number and the percentage of patients with at least one event, categorized by frequency and diagnosis. For hospitalizations, we also determined the average number of admissions and length of stay. Finally, for outpatient services, we reported the absolute number and the percentage of patients from the cohort that accessed each service and the average number of services used.

Healthcare resource costs were valued using the following sources: pharmaceutical costs were based on the gross expenditure for community pharmacy dispensations and the real hospital price (including value-added tax) for drugs supplied through hospital pharmacies. Inpatient hospital costs were valued using diagnosis-related group (DRG) tariffs, which are used by the National Health Service (SSN) for reimbursement. The DRG system provides a bundled payment for the entire hospital stay, from admission to discharge, reflecting the overall complexity of care without itemizing individual procedures. Finally, costs for outpatient diagnostics and procedures (both invasive and noninvasive) were assessed using the current national outpatient tariff schedule. Costs were expressed both as the mean cost per patient using that specific healthcare service or treatment and as the per capita cost for the entire cohort.

### Statistical analysis

Continuous variables are presented as mean ± standard deviation (SD) and median (first and third quartiles). Categorical variables are summarized as frequencies and percentages. Data were extracted by means of Oracle SQL Developer (Italian version 18.1.0.095 and subsequent versions, California, United States). All descriptive analyses were performed using Microsoft Excel for Office 365 (United States) and RStudio (version 2022.07.2, United States), an integrated development environment for the R programming language. The comparisons between the cohort with treated hypoparathyroidism and the sub-cohort with HTI hypoparathyroidism were analyzed using *t*-test or chi-square test, as appropriate for the variable types and distributions. The *t*-test was also used to compare the prevalence values for different years. *P*-values <0.05 have been considered statistically significant.

## Results

### Prevalence of hypoparathyroidism and treated hypoparathyroidism

**Figure 1:**
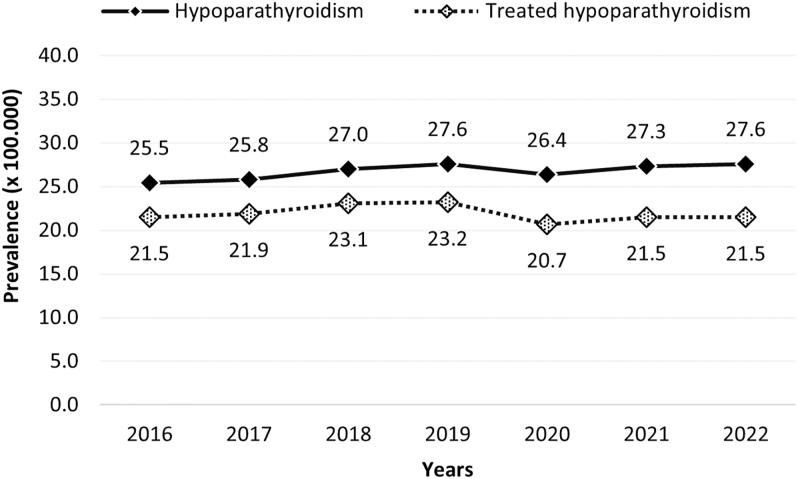
Temporal trend (2016–2022) in the prevalence of hypoparathyroidism and treated hypoparathyroidism.

From 2016 to 2022, the annual number of identified hypoparathyroidism cases ranged from 1,410 to 1,491 within a source population of 5.3 to 5.6 million individuals, representative of the Italian population (Fig. S1 in Supplementary material). This corresponds to an annual estimated prevalence ranging from 25.5 to 27.6 per 100,000 inhabitants (Fig. 1).

Case identification was based on four criteria not mutually exclusive, with their individual contributions detailed below:

- Exemption code: this was the most frequently met criterion, identifying 1,074–1,255 patients per year, covering 76.2–84.2% of identified cases.
- Hospital diagnosis: this criterion identified 61–120 cases per year, 4.3–8.1% of identified cases.
- Anterior neck surgery with calcium treatment: this criterion identified 88–249 patients per year, 6.2–16.7% of identified cases.
- Rare syndromes with calcium dispensation: this criterion identified 13–23 cases per year, 0.9–1.5% of identified cases.

Of the patients affected by hypoparathyroidism, those who received at least one medication for the disease were 1,102–1,262 individuals annually, representing 78–86% of the total identified cohort. The estimated prevalence of treated hypoparathyroidism was 20.7–23.1 per 100,000 inhabitants (Fig. 1).

The prevalence of hypoparathyroidism increased slightly during the study period, rising from 25.5 to 27.6 per 100,000 between 2016 and 2022, with a difference statistically significant (*P* < 0.05). There was a constant increase during this period, except for 2020 when prevalence decreased slightly. The trend in the prevalence of treated patients, after an increase from 2016 to 2019, decreased in 2020 and then remained stable at 21.5 per 100,000 in the past years (Fig. 1).

### Characterization of treated hypoparathyroidism and HTI hypoparathyroidism

Based on the 6-year accrual period, a cohort of 2,791 patients with treated hypoparathyroidism was identified. The demographic characteristics of the cohort are summarized in Table 1. These patients were predominantly female (79.7%), with the highest proportion of patients falling within the age range of 50–64 years (32.5%). The mean age of the cohort was 56 years (±15 SD), with a median of 57 years.

**Table 1:** Demographics and clinical characteristics of patients with treated hypoparathyroidism and HTI hypoparathyroidism.

Characteristics	Cohort with treated hypoparathyroidism	Sub-cohort with HTI hypoparathyroidism
	*n* = 2,791	*n* = 662
Female *n* (%)	2,225 (79.3)	507 (76.6)
Mean age, years ± SD	56 ± 15	54 ± 16
Median age, years (Q1; Q3)	57 (47; 66)	55 (45; 65)
Age (years) group, *n* (*n*/*n*%)*		
<18	40 (1.4)	16 (2.4)^†^
18–34	174 (6.2)	60 (9.1)^†^
35–49	643 (23.0)	170 (25.7)^†^
50–64	1,105 (39.6)	236 (35.6)^†^
65–74	583 (20.9)	124 (18.7)^†^
≥75	246 (8.8)	56 (8.5)^†^
Comorbidities, *n* (*n*/*n*%)		
0	692 (24.8)	141 (21.3)
1	910 (32.6)	224 (33.8)
2	569 (20.4)	154 (23.3)
3	321 (11.5)	71 (10.7)
≥ 4	299 (10.7)	72 (10.8)
Arterial hypertension	1,315 (47.1)	309 (46.7)
Neoplasia	930 (33.3)	288 (43.5)^†^
Thyroid neoplasia	553 (19.8)	209 (31.6)^†^
Dyslipidemia	574 (20.6)	124 (18.7)
Chronic pulmonary disease	364 (11.3)	79 (11.9)
Diabetes	316 (11.3)	70 (10.6)
Depression	250 (9.0)	51 (7.7)
Chronic kidney diseases	239 (8.6)	60 (9.1)
Cardiac arrhythmia	155 (5.6)	47 (7.1)
Heart failure	152 (5.4)	38 (5.7)
Chronic liver diseases	116 (4.2)	32 (4.8)
Cerebral diseases	104 (3.7)	17 (2.6)
Coronary artery diseases	96 (3.4)	15 (2.3)

* *P*-value calculated as comparison between distributions.

^†^ *P*-value <0.05.

Of the patients with treated hypoparathyroidism, 75.2% had at least one chronic condition and 22.2% had three or more comorbidities. The most prevalent condition was arterial hypertension, affecting 47.1% of patients. Neoplasia (a history of, or active disease) was present in 33.3% of patients; specifically, thyroid cancer affected 19.8% of the cohort. Other frequent comorbidities included dyslipidemia (20.6%), chronic lung disease (13.0%), and diabetes (11.3%) (Table 1).

The same analysis was performed on the sub-cohort with HTI hypoparathyroidism. This sub-cohort, identified from the database, comprised 662 patients. A high prevalence of female patients was observed (76.6%). The mean age of this subgroup was 2 years lower than that of patients with treated hypoparathyroidism, with a median age of 55 years (Table 1).

The clinical profile of patients with HTI hypoparathyroidism revealed a high comorbidity burden. In particular, 78.7% of patients had at least one chronic disease and 21.5% had three or more concurrent pathologies. The most frequent condition was arterial hypertension, affecting 46.7% of the sub-cohort, followed by neoplasia (43.5%) and dyslipidemia (18.7%). Furthermore, a history of thyroid cancer was recorded in 31.6% of the sub-cohort.

### Healthcare resource consumption and costs of treated hypoparathyroidism and HTI hypoparathyroidism

Healthcare resource consumption was analyzed for pharmaceuticals, hospitalizations, and outpatient services for the cohort and sub-cohort.

As mandated by the inclusion criteria, all patients received at least one treatment for hypoparathyroidism. The most common dispensations were vitamin D and analogs (89.1%) and calcium salts (77.9%), followed by teriparatide (0.5%). In terms of dosage, 73.2% of treated patients received an average daily dose of 0–2,000 mg of calcium supplements and 66.6% received 0–0.99 μg of calcitriol.

Of the other drugs used for hypoparathyroidism treatment, drugs for hyperphosphatemia were dispensed in 0.3% of the cohort, magnesium in 0.1%, and thiazide diuretics in 15.6% (Table 2).

**Table 2:** Drug dispensations received by patients with treated hypoparathyroidism and HTI hypoparathyroidism. Data are presented as *n* (%).

Hypoparathyroidism treatments	Cohort with treated hypoparathyroidism	Sub-cohort with HTI hypoparathyroidism
	*n* = 2,791	*n* = 662
Vitamin D and analogs	2,487 (89.1)	617 (93.2)
Calcium salts	2,175 (77.9)	601 (90.8)*
Teriparatide	15 (0.5)	15 (2.3)*
Thiazide diuretics	436 (15.6)	107 (16.2)
Drugs for hyperphosphatemia	9 (0.3)	5 (0.8)
Magnesium	2 (0.1)	2 (0.3)

* *P*-value <0.05.

Drugs for other chronic or acute conditions were also frequently dispensed, including antibiotics (63.2%), medications for acid-related disorders (46.8%), anti-inflammatory and antirheumatic products (43.7%), and cardiovascular drugs, especially agents acting on the renin–angiotensin system (35.9%) (Table S5 in Supplementary Material).

During the 12-month follow-up, 24.2% of patients with treated hypoparathyroidism were hospitalized at least once (mean: 1.4 admissions). Most hospitalizations were for procedures and conditions associated with hypoparathyroidism, such as radiotherapy involving 8.2% of the cohort and thyroid cancer involving 1.4%. Admissions specifically for hypocalcemia or nephrocalcinosis were less common, involving 1% of patients (Table S5 in Supplementary Material).

A total of 24.7% of patients visited the ED at least once during the follow-up (mean: 1.7 access); notably, 1.2% of the cohort presented with hypocalcemia, and 32.6% of these hypocalcemia cases required subsequent hospitalization (Table S5 in Supplementary Material).

Finally, during the 12-month follow-up, 94.6% of the cohort utilized at least one outpatient diagnostic or specialist service. Laboratory testing was the most frequent service (88.0% of patients). Among these, plasma calcium level testing was the most common (73.9% of the cohort), with an average of 4.3 tests per patient per year. Vitamin D and phosphorus levels were tested in 43.4 and 42.4% of patients, with annual averages of 1.9 and 2.6 tests, respectively. Endocrinological consultations were required by 19.4% of patients (Table S5 in Supplementary Material).

The same analysis was performed on the sub-cohort with HTI hypoparathyroidism monitoring and observing pharmaceuticals, hospitalization, and outpatient services. Regarding pharmaceutical dispensations, the most common medications were vitamin D and analogs (93.2%), mineral supplements containing calcium salts (90.8%), and thyroid treatments (86.7%). In contrast, other hypoparathyroidism-specific medications, such as thiazides, teriparatide, and drugs for hyperphosphatemia, were rarely dispensed (Table 2). Nearly all patients (99.4%) received at least one medication for comorbidities, most frequently antibiotics (67.4%), drugs for acid-related disorders (50.8%), and anti-inflammatory/antirheumatic products (40.6%) (Table S5 in Supplementary Material).

For hospital care, 37.8% of the sub-cohort experienced at least one hospitalization (average 1.5 admissions), with 15.7% of the entire sub-cohort being admitted for radiotherapy and 2.9% for thyroid malignancy. Moreover, 2.7% were hospitalized for hypocalcemia, and less than 1% for nephrocalcinosis or tetany. ED access was also frequent, with 32.5% of the cohort having at least 2 accesses. Hypocalcemia was the most common primary diagnosis for these accesses, involving 3.5% of the sub-cohort, and 35.5% of these hypocalcemia cases required subsequent hospitalization.

Outpatient service utilization was nearly universal, with 98.2% of patients requiring at least one specialist examination or diagnostic service. Laboratory testing was predominant: 94.6% of patients had plasma calcium levels monitored, with an average of 8.2 tests per patient per year. Testing for phosphorus (66.8%) and vitamin D (60.9%) was less frequent. Endocrinological examinations were required by 24.0% of the sub-cohort.

The direct economic burden was evaluated for both cohort and sub-cohort. In the treated hypoparathyroidism cohort, the mean annual per capita expenditure charged to the SSN was €2,885. The cost distribution was 37.3% for pharmaceuticals, 44.9% for hospitalizations, and 17.7% for outpatient services (Table 3).

**Table 3:** Per-patient mean direct costs charged to the Italian National Health Service (SSN) during the follow-up year for patients with treated hypoparathyroidism and HTI hypoparathyroidism.

Administrative data flow	Per-patient mean costs	
	Cohort with treated hypoparathyroidism	Sub-cohort with HTI hypoparathyroidism
	€ (%)	€ (%)
Pharmaceuticals	1,077 (37.3)	1,273 (32.0)
Vitamin D and analogs	87 (3.0)	120 (3.0)
Calcium salts	51 (1.8)	85 (2.2)
Teriparatide	31 (1.1)	131 (3.3)
Thiazide diuretics	15 (0.5)	13 (0.3)
Magnesium	<1 (<0.1)	<1 (<0.1)
Drugs for hyperphosphatemia	3 (0.1)	7 (0.2)
Hospitalization	1,297 (44.9)	1,725 (43.4)
Outpatient services	511 (17.7)	974 (24.5)
Total	2,885	3,522

In the HTI hypoparathyroidism sub-cohort, the mean annual per capita cost was higher, at €3,971. The cost distribution for this group was 32.0% for pharmaceuticals, 43.4% for hospitalizations, and 24.5% for outpatient services (Table 3).

## Discussion

This study provides a comprehensive, real-world characterization of the clinical and epidemiological profile of hypoparathyroidism in Italy, utilizing a large administrative database. The estimated prevalence of 27 per 100,000 individuals aligns with prior Italian work (30) and international reports (22–37 per 100,000) (19, 20), confirming the disorder’s rarity. The studied cohort (i.e. patients with treated hypoparathyroidism) was characterized by a high prevalence of women, especially in middle-aged, and a high comorbidity burden. This clinical profile indicates that hypoparathyroidism is not an isolated endocrine disorder, but it is a condition embedded within a complex multimorbid framework, which consequently drives substantial healthcare resource utilization.

The therapeutic management was performed primarily with calcium salts and calcitriol, whereas other therapies, including thiazide diuretics, teriparatide, and magnesium supplements, were used in low percentages (31). Teriparatide, a PTH analog approved for the treatment of osteoporosis but reimbursed off-label in Italy under a special funding in refractory cases (law 648/96), was used in negligible proportion of patients, a finding that may be influenced by its off-label status, subcutaneous route of administration requiring multiple daily injections, unproved long-term safety, and overall efficacy in management of hypoparathyroidism (32). Magnesium supplements, which are specifically indicated for correcting concomitant hypomagnesemia, were administered in rare cases (33); this is certainly underestimated since these drugs, in most cases, are not reimbursed by the SSN and are, therefore, not recorded in administrative databases. Collectively, these prescription patterns have the potential to indicate a discrepancy between clinical ESE guidelines (12) and real-world practice in Italy. This discrepancy suggests the need to have more specific treatment strategies.

Healthcare consumption of patients with treated hypoparathyroidism was marked by significant hospital and ED utilization, as reported in other studies (34, 35). Furthermore, significant use of antibacterials and anti-inflammatory agents was observed among patients in the cohort and sub-cohort, in agreement with a previous study that reported a higher risk of infections for these subjects (36) and with the prescription pattern observed in patients with chronic hypoparathyroidism in Sweden (37).

These findings were even more pronounced for the sub-cohort with HTI disease, which exhibited a distinctly greater clinical and economic burden. In fact, although the aim of the research was not a formal comparative analysis between HTI and treated patients, the obtained data offer the opportunity to observe some differences between the cohort and sub-cohort. The sub-cohort with HTI hypoparathyroidism showed higher comorbidity rates (78.7% with ≥1 condition), markedly higher dispensation of calcium salts (90.8%), more frequent hospitalizations and ED visits for disease-related complications (9.6 and 19.2%, respectively), and intensified laboratory monitoring, compared with the entire cohort with treated disease. In contrast, calcitriol prescriptions were only slightly increased in the sub-cohort (93.2%). Of note, calcium supplementation itself may contribute to complications and healthcare utilization, independently of disease severity in patients with hypoparathyroidism (38). Regarding age, the subgroup was on average 2 years younger than the treated cohort, thereby possibly reflecting a higher proportion of idiopathic cases in the sub-cohort and postsurgical cases in the main cohort. However, this hypothesis is difficult to demonstrate without ascertaining diagnoses.

Interpretation of these findings must consider the strengths and limitations of the administrative data source. In fact, the primary strength of this analysis lies in the use of the ReS database, which includes real-world data from over 5 million SSN beneficiaries, representing approximately 9% of the Italian population. The database’s demographic overlaps with the national population. This study successfully identified a substantial number of patients and achieved its aim of comprehensively describing the direct healthcare burden, demographics, and comorbidity profile associated with this condition.

However, several limitations must be considered. As the analysis relies on the secondary use of data capturing only services reimbursed by the SSN, it is subject to all the well-known limitations of healthcare administrative data (39). The main limitation of the study was the potential for misclassification when identifying hypoparathyroidism using proxies, due to the absence of biochemical measurements. This limitation particularly affected the criterion based on anterior neck surgery, which was consistent with other studies (21) and had only a slight impact on prevalence estimates. However, focusing the analysis only on treated patients could have mitigated this limitation. Moreover, lack of detailed clinical data (e.g., laboratory values such as serum calcium or PTH levels), primary care electronic health records, and information on patients’ out-of-pocket expenses, which constitute a significant portion of pharmaceutical spending in Italy (40), could have affected the study results. Among limitations, it is imperative to acknowledge the utilization of dispensation data that do not ensure the assumption of drugs by the patient; this is of particular significance in the definition of the HTI sub-cohort.

As such, this study is intended to be purely descriptive; the above-mentioned limitations preclude definitive conclusions on disease control or relationships between hypoparathyroidism and other variables, such as comorbidities.

## Conclusion

This study successfully characterized the epidemiological and clinical profile of hypoparathyroidism in Italy. Our findings demonstrate that the disease carries a high burden of comorbidities and direct costs, which are markedly higher in patients requiring intensive therapy (HTI).

These results highlight an unmet need, confirming that patients requiring HTI represent a priority target for optimized management and the implementation of more specific therapies.

## Supplementary materials



## Declaration of interest

LD, NA, LeonardoD, GR, ID, CP, and NM are employees of Fondazione ReS and have no competing interests with any financial organizations regarding the materials discussed in the manuscript. AA and IE are employees of Drugs and Health Srl and have no competing interests with any financial organizations regarding the materials discussed in the manuscript. AP is a consultant for Theramex, Bruno Farmaceutici, and Amgen. AP receives research funding from Amgen, Shire, and Ascendis; is in the speakers bureau of UCB and Amgen; and receives an industry grant from Amgen. GZ declares that he received honoraria and consulting fees from Ascendis Pharma. APiazza declares that she has no conflict of interest. ES declares that she received honoraria and consulting fees from Ascendis Pharma.

## Funding

This work was supported by unconditional funding from Ascendis Pharma. The financial support for this study was provided with a funding agreement ensuring maintenance of author independence in study design, data interpretation, writing, and decisions to publish.
