# Supplementary material for: Hypoparathyroidism: clinical profiles, healthcare use, and costs from real-world data from Italy
Source: Endocr Connect. 2026 Mar 26;15(3):e250844. doi: 10.1530/EC-25-0844 (PMC13052763; doi:10.1530/EC-25-0844)
Supplement: Supplementary file 1 [file supplementary_materials.pdf]

Figure S1 - Percentage distribution of the Italian population in the Fondazione Ricerca e Salute (ReS) database and according to the Italian Institute of Statistics (ISTAT), by age group

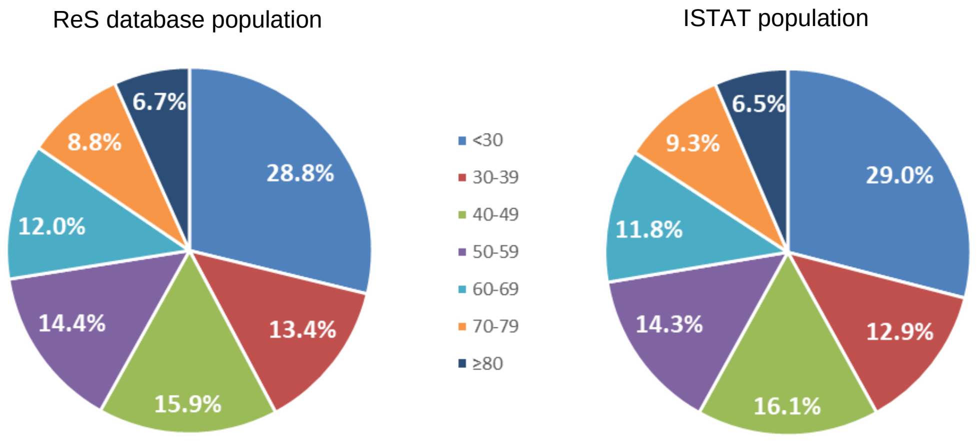

Table S1. List of codes used for hypoparathyroidism identification criteria

Administrative data flow	Codes* and descriptions
Exemption criterion	
Exemptions	026.252.1 - hypoparathyroidism
**Hospitalization criterion**	
Hospitalizations (diagnosis)	252.1 - Hypoparathyroidism
Surgery + (Diagnosis or Drugs) criterion	
Hospitalizations (procedures)	06.2 - Unilateral thyroid lobectomy
	06.9. - Other partial thyroidectomy
	06.4 - Complete thyroidectomy
	06.5x - Substernal thyroidectomy
	06.6 - Excision of lingual thyroid
	06.8x - Parathyroidectomy
	06.9x - Other operations on thyroid (region) and parathyroid
	30.3 - Complete laryngectomy
	30.4 - Radical laryngectomy
Hospitalizations (diagnosis)	275.41 - Hypocalcemia
	781.7 - Tetany
Pharmaceutical	A12AA - Calcium
	A12AX - Calcium, combinations with vitamin D and/or other drugs
Rare syndromes + Drug criterion	
Hospitalizations (diagnosis)	279.11 - DiGeorge syndrome
	775.4 - Hypocalcemia and hypomagnesemia of newborn
Exemptions	RCG030 - Autoimmune polyendocrinopathy

	RN0850 - CHARGE syndrome
	RCG103 - Other metabolic congenital disorders associated with metal transport and metabolism
	RCG160 - Primary immunodeficiency diseases (Agammaglobulinemia, DiGeorge syndrome, Nijmegen syndrome)
	RC0300 – Kenny-Caffey Syndrome
Pharmaceutical	A12AA - Calcium
	A12AX - Calcium, combinations with vitamin D and/or other drugs

ICD9 CM (International Classification of Diseases, 9th revision - Clinical Modification) codes for hospitalizations; ATC (Anatomical Therapeutic Chemical) codes for drugs; National exemption codes for exemptions

Table S2. List of ATC codes of drugs for hypoparathyroidism treatment

**Administrative data flow**	**ATC Codes and descriptions**
Pharmaceuticals	H05AA02 - Teriparatide
	**Calcium**<br>A12AA - Calcium<br>A12AX - Calcium, combinations with vitamin D and/or other drugs
	**Vitamin D and analogues**<br>A11CC03 - Alfacalcidol<br>A11CC04 - Calcitriol<br>A11CC05 - Colecalciferol<br>M05BB03 - Alendronic acid and colecalciferol

ATC: Anatomical Therapeutic Chemical codes for drugs

Table S3: Criteria and codes identifying comorbidities

Diabetes mellitus		
**At least one hospital discharge form with primary/secondary diagnosis (ICD-9-CM code)**	**Disease waiver claim**	**At least 2 drug supplies within one year before the index date (ATC codes)**
250.* – Diabetes mellitus	013 – Diabetes mellitus	A10 – Drugs used in diabetes
**Dyslipidemia**		
**At least one hospital discharge form with primary/secondary diagnosis (ICD-9-CM code)**	**Disease waiver claim**	**At least 2 drug supplies within one year before the index date (ATC codes)**
272.* - Disorders of lipoid metabolism	025 - Type IIa and IIb heterozygous familial hypercholesterolemia – Polygenic hypercholesterolemia – Familial combined hypercholesterolemia – Type III hyperlipoproteinemia	C10A – Lipid modifying agents, plain<br>C10B - Lipid modifying agents, combinations
**Arterial hypertension**		
**At least one hospital discharge form with primary/secondary diagnosis (ICD-9-CM code)**	**Disease waiver claim**	**At least 2 drug supplies within one year before the index date (ATC codes)**
401.* – Essential hypertension<br>402.* – Hypertensive heart disease<br>403.* – Hypertensive chronic kidney disease<br>404.* – Hypertensive heart and chronic kidney disease<br>405.* - Secondary hypertension	031 – Arterial hypertension<br>0A31 - Arterial hypertension without organ damage	C02 – Antihypertensives<br>C03 – Diuretics<br>C07 – Beta blocking agents<br>C08 – Calcium channel blockers<br>C09 – Agents acting on the renin-angiotensin system
**Heart failure**		
**At least one hospital discharge form with primary/secondary diagnosis (ICD-9-CM code)**	**Disease waiver claim**	**At least 2 drug supplies within one year before the index date (ATC codes)**
402.01 – Malignant hypertensive heart disease with heart failure<br>402.11 – Benign hypertensive heart disease with heart failure<br>402.91 – Unspecified hypertensive heart disease with heart failure<br>404.01 – Hypertensive heart and chronic kidney disease, malignant, with heart failure and with chronic kidney disease stage I through stage IV, or unspecified<br>404.03 – Hypertensive heart and chronic kidney disease, malignant, with heart failure and with chronic kidney disease stage V or end stage renal disease<br>404.11 – Hypertensive heart and chronic kidney disease, benign, with heart failure and with chronic kidney disease stage I through stage IV, or unspecified<br>404.13 – Hypertensive heart and	021 – Heart failure	C09DX04 – Valsartan and sacubitril

chronic kidney disease, benign, with heart failure and chronic kidney disease stage V or end stage renal disease<br>404.91 – Hypertensive heart and chronic kidney disease, unspecified, with heart failure and with chronic kidney disease stage I through stage IV, or unspecified<br>404.93 – Hypertensive heart and chronic kidney disease, unspecified, with heart failure and chronic kidney disease stage V or end stage renal disease<br>428.* - Heart failure		
**Chronic kidney disease**		
**At least one hospital discharge form with primary/secondary diagnosis or procedure**	**Disease waiver claim**	**At least one local outpatient specialist service (national tariffs)**
**Primary/secondary diagnosis (ICD-9-CM code)**<br>250.4 – Diabetes with renal manifestations<br>403.* – Hypertensive chronic kidney disease<br>404.* – Hypertensive heart and chronic kidney disease<br>582.* – Chronic glomerulonephritis<br>583.* – Nephritis and nephropathy not specified as acute or chronic<br>585.* – Chronic renal failure<br>586.* – Renal failure unspecified<br>587.* – Renal sclerosis unspecified<br>588.* – Disorders resulting from impaired renal function<br>590.* – Infections of kidney<br>V42.0 – Organ or tissue replaced by transplant<br>V56.* – Encounter for dialysis and dialysis catheter care<br><br>**Primary/secondary procedure (ICD-9-CM code)**<br>39.95 – Hemodialysis<br>54.98 – Peritoneal dialysis<br>55.6x – Renal transplantation	023 – Chronic renal failure<br>061 – Chronic kidney disease<br>052.V42.0 – Renal transplantation	39.95 – Hemodialysis<br>54.98 - Peritoneal dialysis
**Cardiac arrythmias**		
**At least one hospital discharge form with primary/secondary diagnosis (ICD-9-CM code)**	**Disease waiver claim**	**At least 2 drug supplies within one year before the index date (ATC codes)**
426.* – Conduction disorders<br>427.* - Cardiac dysrhythmias	002.426; 0A02.426 – Conduction disorders<br>002.427; 0A02.427 - Cardiac dysrhythmias	C01B – Antiarrhythmics, Class I and III
**Depression**		

At least one hospital discharge form with primary/secondary diagnosis (ICD-9-CM code)	Disease waiver claim	At least 2 drug supplies within one year before the index date (ATC codes)
296.2x - Major depression, single episode<br>296.3x - Major depression, recurring episode<br>296.5x – Bipolar disorder type I, most recent (or current) depressive episode<br>296.82 – Atypical depressive disorder<br>298.0x – Depressive psychosis<br>300.4 - Dysthymic disorder<br>301.12 - Chronic depressive personality disorder	044.296.2 - Psychosis (major depression, single episode)<br>044.296.3 - Psychosis (major depression, recurring episode)<br>044.296.5 - Psychosis (bipolar affective syndrome, depressive episode)<br>044.296.8 - Psychosis (manic-depressive disorder)<br>044.298.0 – Psychosis (depressive psychosis)	N06A - Antidepressants
**Chronic liver diseases**		
**At least one hospital discharge form with primary/secondary diagnosis (ICD-9-CM code)**	**Disease waiver claim**	**At least 2 drug supplies within one year before the index date (ATC codes)**
070.* - Viral hepatitis<br>571.* - Chronic liver disease and cirrhosis<br>572.* - Liver abscess and sequelae of chronic liver disease<br>573.* - Other disorders of liver<br>V42.7 - Liver replaced by transplant	016 – Chronic hepatitis (active)<br>008 – Hepatic cirrhosis, biliary cirrhosis	J05AP - Antivirals for treatment of HCV infections<br>J05AF08 - adefovir dipivoxil<br>J05AF10 - entecavir<br>J05AF11 - telbivudine

**Neoplasia (current or history)**			
**At least one hospital discharge form with**	**Disease waiver claim**	**At least one local outpatient specialist service (national tariffs)**	**At least one drug supplies (ATC codes)**
**Primary/secondary diagnosis (ICD-9-CM code)** 140.* - 208.* - Neoplasms<br>V10.* - Personal history of malignant neoplasm<br>V58.0 - Radiotherapy<br>V58.1x – Chemotherapy<br>AND / OR<br>**Primary/secondary intervention or procedure (ICD-9-CM code)**<br>00.10 - Implantation of chemotherapeutic agents<br>92.2x - Therapeutic radiology and nuclear medicine<br>92.3x – Stereotactic radiosurgery as antineoplastic agents<br>99.25 - Injection or infusion of chemotherapy substances for cancer<br>99.28 - Injection or infusion of biological response modifying agents (BRM)	048 – Malignant neoplasms and tumors of uncertain behavior	92.2x - Therapeutic radiology and nuclear medicine<br>99.24.1 - Infusion of hormonal substances<br>99.25 - Injection or infusion of chemotherapy substances for cancer<br>92.28.6- palliative pain therapy from bone metastases	L01 - Antineoplastic agents

Thyroid diseases

At least one hospital discharge form with primary/secondary diagnosis (ICD-9-CM code)	Disease waiver claim	At least 2 drug supplies within one year before the index date (ATC codes)
242.* - Thyrotoxicosis with or without goiter<br>243.* - Congenital hypothyroidism<br>244.* - Acquired hypothyroidism<br>245.* - Thyroiditis<br>246.* - Other disorders of thyroid	056 - Hashimoto thyroiditis<br>027 - Congenital, severe acquired hypothyroidism<br>035 - Graves' Disease Basedow, other forms of hyperthyroidism	H03AA01 - Levothyroxine sodium<br>H03BB02 - Thiamazole

Cerebrovascular disease

At least one hospital discharge form with primary/secondary diagnosis (ICD-9-CM code)	Disease waiver claim
430 – 438.* - Cerebrovascular Disease	002.433; 0B02.433 - Occlusion and stenosis of precerebral arteries<br>002.434; 0B02.434 - Occlusion of cerebral arteries<br>002.437; 0B02.437 - Other and ill-defined cerebrovascular disease

Coronary artery disease

At least one hospital discharge form with	Disease waiver claim
**Primary/secondary diagnosis (ICD-9-CM code)**<br>410.* - Acute myocardial infarction<br>411.* - Other acute and subacute forms of ischemic heart disease<br>412 - Old myocardial infarction<br>413.* - Angina pectoris<br>414.* - Other forms of chronic ischemic heart disease<br>AND / OR<br>**Primary/secondary intervention or procedure (ICD-9-CM code)**<br>36.* – Operations on vessels of heart<br>00.66 – Percutaneous Transluminal Coronary Angioplasty (PTCA) or coronary atherectomy	(002.414) 0A02.414 – Diseases of the circulatory system

ICD9 CM (International Classification of Diseases, 9th revision - Clinical Modification) codes for hospitalizations; ATC (Anatomical Therapeutic Chemical) codes for drugs; National exemption codes for exemptions

Table S4: List of ATC codes of other drugs for hypoparathyroidism treatment.

Administrative data flow	Codes and descriptions
	**Hyperphosphatemia**<br>V03AE02 - Sevelamer<br>V03AE03 - Lanthanum carbonate<br>V03AE04 - Calcium acetate and magnesium carbonate<br>V03AE05 – Sucroferric oxyhydroxide
	**Magnesium**<br>A12CC - Magnesium<br>B05XA05 - Magnesium sulfate<br>B05XA10 - Magnesium phosphate<br>B05XA11 - Magnesium chloride<br>V03AE04 - Calcium acetate and magnesium carbonate
	**Diuretics thiazides**<br>C03AA - Thiazides, plain<br>C03AB - Thiazides and potassium in combination<br>C03EA01 - Hydrochlorothiazide and potassium-sparing agents<br>C03EA02 - Trichlormethiazide and potassium-sparing agents<br>C03EA05 - Mebutizide and potassium-sparing agents<br>C03EA07 - Cyclopenthiazide and potassium-sparing agents<br>C03EA13 - Bendroflumethiazide and potassium-sparing agents<br>C09BA01 - Captopril and diuretics<br>C09BA02 - Enalapril and diuretics<br>C09BA03 - Lisinopril and diuretics<br>C09BA05 - Ramipril and diuretics<br>C09BA06 - Quinapril and diuretics<br>C09BA07 - Benazepril and diuretics<br>C09BA08 - Cilazapril and diuretics<br>C09BA09 - Fosinopril and diuretics<br>C09BA15 - Zofenopril and diuretics<br>C09BX03 - Ramipril, amlodipine and hydrochlorothiazide<br>C09DA - Angiotensin II receptor blockers (ARBs) and diuretics<br>C09DX01 - Valsartan, amlodipine and hydrochlorothiazide<br>C09DX03 Olmesartan medoxomil, amlodipine and hydrochlorothiazide<br>C09DX06 - Candesartan, amlodipine and hydrochlorothiazide<br>C09DX07 - Irbesartan, amlodipine and hydrochlorothiazide<br>C09DX08 - Telmisartan, amlodipine and hydrochlorothiazide<br>C09XA52 - Aliskiren and hydrochlorothiazide<br>C09XA54 - Aliskiren, amlodipine and hydrochlorothiazide

ATC: Anatomical Therapeutic Chemical codes for drugs

Table S5. Top 10s pharmaceutical dispensations, hospitalizations, accesses to Emergency Department and outpatient services of patients with treated hypoparathyroidism and severe hypoparathyroidism.

**Healthcare Resources**	**Cohort with treated hypoparathyroidism** *N=2791*	**Sub-cohort with severe hypopara-thyroidism** *N=662*
**Pharmaceuticals**	n (%)	n (%)
Vitamins	2569 (92.0)	630 (95.2)
Mineral supplements	2305 (82.6)	601 (90.8)
Thyroid therapy	2179 (78.1)	574 (86.7)
Antibacterials for systemic use	1765 (63.2)	446 (67.4)
Medicines for acid-related disor-ders	1306 (46.8)	336 (50.8)
Anti-inflammatory and an-tirheumatic products	1219 (43.7)	269 (40.6)
Agents acting on the renin-angiotensin system	1003 (35.9)	230 (34.7)
Corticosteroids for systemic use	733 (26.3)	208 (31.4) *
Antithrombotic agents	724 (25.9)	178 (26.9)
Beta blocking agents	687 (24.6)	163 (24.6)
**Hospitalizations**	n (%)	n (%)
Radiotherapy	229 (8.2)	104 (15.7) *
Thyroid malignancy (anamnesis)	39 (1.4)	19 (2.9) *
Hypocalcemia	24 (0.9)	18 (2.7) *
Malignant neoplasm of thyroid gland	12 (0.4)	4 (0.6)
Acute respiratory failure	12 (0.4)	/
Hypoparathyroidism	10 (0.4)	7 (1.1) *
Secondary and unspecified ma-lignant neoplasm of lymph nodes of head, face, and neck	9 (0.3)	4 (0.6)
Acute kidney failure, unspecified	9 (0.3)	4 (0.6)
Postsurgical hypoparathyroidism	8 (0.3)	3 (0.5)
Renal calcinosis	7 (0.3)	4 (0.6)
At least one recovery	675 (24.2)	250 (37.8) *
**ED Accesses**	n (%)	n (%)
Other signs and symptoms in breast	38 (1.4)	13 (2.0)
Other specified congenital anom-alies	37 (1.3)	10 (1.5)
Hypocalcemia	34 (1.2)	23 (3.5) *
Other malaise and fatigue	33 (1.2)	18 (2.7)
Other general symptoms	27 (1.0)	7 (1.1)
Other unknown and unspecified cause of morbidity and mortality	25 (0.9)	10 (1.5)
Other chest pain	23 (0.8)	9 (1.4)
Generalized abdominal pain	21 (0.8)	11 (1.7) *
Appendicular appendiceal colic	20 (0.7)	/
Nephrocalcinosis	16 (0.6)	14 (2.1) *
Electrolyte and fluid disorders not elsewhere classified	/	9 (1.4)

At least one access	690 (24.7)	215 (32.2) *
**Outpatient services**	n (%)	n (%)
Laboratory tests	2457 (88)	640 (96.7)
Total calcium	2063 (73.9)	626 (94.6)
Vitamin D	1210 (43.4)	442 (66.8) *
Phosphorous	1183 (42.4)	403 (60.9) *
NS specialistic visit	778 (27.9)	266 (40.2) *
Thyroid region diagnostic imaging	644 (23.1)	236 (35.6) *
Endocrinology visit	542 (19.4)	164 (24.8) *
ECG	498 (17.8)	159 (24.0) *
Abdomen diagnostic imaging	438 (15.7)	148 (22.4) *
Breast diagnostic imaging	342 (12.3)	299 (45.2) *
Muscle-skeletal diagnostic imaging	340 (12.2)	321 (48.5) *
Chest diagnostic imaging	321 (11.5)	340 (51.4) *
Cardiologic visit	299 (10.7)	/
At least one outpatient service	2641 (94.6)	650 (98.2)

*p>0.05
